# High-Dose Intravenous Ferric Carboxymaltose/Derisomaltose Without ESAs for Cancer-Related Anemia in Japan: A Retrospective Single-Center Cohort Study

**DOI:** 10.3390/cancers18030416

**Published:** 2026-01-28

**Authors:** Shinya Kajiura, Yudai Ishikawa, Yoko Mizuno, Akihiro Yoshida, Ryutatsu Yuki, Toshihito Horikawa, Mutsuki Furukawa, Kohei Nagata, Iori Motoo, Takayuki Ando, Ichiro Yasuda, Atsushi Kato, Ryuji Hayashi

**Affiliations:** 1 Department of Medical Oncology and Palliative Medicine, Toyama University Hospital, Toyama 930-0194, Japan; mizunoy@med.u-toyama.ac.jp (Y.M.); ayoshida@med.u-toyama.ac.jp (A.Y.); horihori@med.u-toyama.ac.jp (T.H.); mutsu555@med.u-toyama.ac.jp (M.F.); hsayaka@med.u-toyama.ac.jp (R.H.); 2 Palliative Care Team, Toyama University Hospital, Toyama 930-0194, Japan; ryuki@med.u-toyama.ac.jp; 3 Third Department of Internal Medicine, University of Toyama, Toyama 930-0194, Japan; nagatako59@gmail.com (K.N.); iori4869@med.u-toyama.ac.jp (I.M.); takayuki@med.u-toyama.ac.jp (T.A.); yasudaic@med.u-toyama.ac.jp (I.Y.); kato@med.u-toyama.ac.jp (A.K.); 4 Department of Hospital Pharmacy, Toyama University Hospital, Toyama 930-0194, Japan; s0760257_pha@yahoo.co.jp

**Keywords:** cancer-related anemia, intravenous iron, ferric carboxymaltose, ferric derisomaltose, functional iron deficiency, transfusion avoidance, hypophosphatemia, supportive care, real-world evidence, oncology

## Abstract

**Background/Objectives:** In Japan, cancer-related anemia (CRA) is common, and erythropoiesis-stimulating agents (ESAs) are not approved for chemotherapy-induced anemia. Modern intravenous (IV) iron formulations, such as ferric carboxymaltose (FCM) and ferric derisomaltose (FDI), enable high-dose repletion; however, real-world evidence in ESA-free oncology settings remains limited. **Methods:** This single-center retrospective study included patients with CRA (*N* = 55) who received high-dose IV iron (FCM or FDI). Iron phenotypes were classified as absolute iron deficiency (ID), functional ID, or non-ID. The primary endpoint was hemoglobin (Hb) change from baseline to approximately 1 month (21–45 days) in the non-transfused patients. Secondary endpoints included responder rate (ΔHb ≥ 1.0 g/dL), transfusion avoidance rate, dosing adequacy relative to Ganzoni-calculated iron deficit, and safety, particularly hypophosphatemia. **Results:** Among the non-transfused patients, mean Hb increased from 8.76 ± 1.34 g/dL to 9.73 ± 1.75 g/dL (mean ΔHb +0.92 ± 1.44 g/dL; *p* < 0.001). The responder and transfusion avoidance rates were 48.9% and 81.8%, respectively. Functional ID was most prevalent (52.7%), with clinically meaningful Hb responses. A total of 38.2% achieved approximately 1000 mg dosing. The safety profile was excellent, and no infusion reactions or symptomatic hypophosphatemia was observed (median serum phosphate changed from 3.4 [3.0–3.9] to 3.2 [2.7–3.8] mg/dL). **Conclusions:** In this real-world Japanese oncology setting where ESAs were not available for chemotherapy-induced anemia, high-dose IV iron monotherapy (FCM or FDI) was well tolerated and was associated with modest short-term Hb increases and a high observed rate of transfusion avoidance within a 21–45-day assessment window. These findings suggest that a proactive, TSAT-guided IV iron therapy approach may be a pragmatic option for selected patients; however, durability beyond 1 month, optimal re-dosing, and generalizability require confirmation in larger, longer prospective studies.

## Simple Summary

In Japan, erythropoiesis-stimulating agents (ESAs) are not approved for chemotherapy-induced anemia; hence, many patients rely on transfusions. In this real-world data study, we evaluated the newest high-dose intravenous iron (ferric carboxymaltose or ferric derisomaltose) formulations in routine oncology practice. In 55 patients with cancer-related anemia, hemoglobin increased by approximately 1.2 g/dL at 1 month; 50% of the patients achieved a clinically meaningful rise (≥1 g/dL), and 82% avoided transfusion. Functional iron deficiency was the most common phenotype and responded well to treatment. The safety profile was excellent, with no infusion-related reactions or symptomatic hypophosphatemia (only a slight median change in serum phosphate levels). We further developed a transferrin saturation (TSAT)-guided clinical pathway to guide patient and dosing selection. Proactive, TSAT-guided intravenous iron may be a practical, ESA-free option to manage cancer-related anemia and reduce transfusion dependence in Japan.

## 1. Introduction

Cancer-related anemia (CRA), a common complication of both solid and hematologic malignancies, is often exacerbated by cytotoxic chemotherapy. It is strongly associated with fatigue, reduced functional capacity, impaired quality of life (QoL), and even poor treatment response and survival [1]. The multifactorial pathogenesis of CRA includes blood loss, nutritional deficiencies, *functional iron deficiency* (ID), wherein inflammatory cytokines (e.g., via hepcidin) cause iron sequestration and impaired iron utilization despite normal or elevated ferritin stores [1], and *absolute* ID (depleted ferritin stores), especially in patients with chronic bleeding or malnutrition. In clinical terms, absolute iron deficiency is defined by low ferritin (<100 ng/mL) levels with transferrin saturation (TSAT) < 20%, and functional iron deficiency by normal or high ferritin (≥100 ng/mL) levels with TSAT < 20% [2]. Both forms are prevalent in patients with CRA [3], highlighting the role of iron-restricted erythropoiesis as a major driver of CRA.

International guidelines, such as those by the American Society of Clinical Oncology/American Society of Hematology, European Society for Medical Oncology (ESMO), and National Comprehensive Cancer Network, recommend three main approaches for addressing CRA: red blood cell (RBC) transfusion for immediate correction, erythropoiesis-stimulating agents (ESAs) for chemotherapy-induced anemia, and iron supplementation for iron deficiency [2,4,5]. These guidelines conventionally recommend initiating ESAs in palliative chemotherapy recipients who have symptomatic anemia (hemoglobin [Hb] of <10 g/dL) [4]. Although ESAs can reduce transfusion needs, safety concerns (e.g., thromboembolism and potential negative impact on survival) prompt conservative use, particularly in non-curative settings, targeting an Hb level adequate enough to avoid transfusions [4]. Crucially, the guidelines recommend evaluating iron status in patients with anemia and treating any ID before or alongside ESA administration [4]. Intravenous (IV) iron-supplemented ESA therapy has been reported to improve hematopoietic response by 29% and decrease transfusion risk by 23% compared with ESAs alone [6,7]. Thus, iron supplementation has been strongly recommended for patients with cancer receiving ESAs, if any ID is present [4]. Notably, current ESMO guidelines recommend considering IV iron alone for patients with functional ID receiving chemotherapy [2], reflecting the accumulating evidence on the erythropoietic ability of IV iron in CRA.

CRA management in Japan differs significantly from that in Western countries. ESAs are not approved for chemotherapy-induced anemia (CIA) in Japan, and hence, rarely used in routine oncology care [8]. Approximately 70% of chemotherapy recipients in Japan develop anemia (Hb < 10 and <8 g/dL in 40% and 23%, respectively), with only 16% receiving RBC transfusions [8]. Sans ESAs, many patients with moderate anemia are managed with observation and oral iron, or eventual transfusion for severe anemia. Oral iron is the widely used first-line therapy for ID anemia in Japan owing to its low cost and convenience [9]. However, oral iron causes gastrointestinal side effects in 10–20% of patients and requires prolonged administration (≥3 months) to replete iron stores; hence, its efficacy in oncology settings is limited [1,9]. Moreover, because oral iron is often ineffective during active inflammation, guidelines recommend that it should only be considered in absolute ID without inflammation (e.g., normal C-reactive protein [CRP] levels) [2], which is relatively uncommon in cancer. Nevertheless, the lack of CRA guidelines in the Japanese context and the unavailability of modern IV iron formulations drive the continued reliance on oral iron or transfusion in practice.

Internationally. IV iron supplementation has emerged as a preferred approach for treating ID-related CRA, especially in patients who cannot or will not use ESAs. Ferric carboxymaltose (FCM) and ferric derisomaltose (FDI, or iron isomaltoside 1000), the two “third-generation” IV iron formulations currently available in Japan, permit much higher single doses than older formulations (saccharated ferric oxide [SFO]; 40–120 mg [10]). FCM can be administered as a single infusion of up to 500 mg (or 750–1000 mg depending on country-specific labels) and FDI as a 1000 mg infusion, facilitating rapid delivery of the total iron needed. Accordingly, international guidelines have shifted toward fixed high-dose IV iron for patients with cancer with functional ID. For example, the ESMO guidelines recommend an IV iron dose of 1000 mg in patients with functional ID and an Hb of 8–10 g/dL [2]. A single 1000 mg infusion (FCM or FDI) can significantly improve Hb and reduce transfusion requirements in patients with CRA [7]. Notably, approximately 50% of patients with CRA are estimated to require iron supplementation, and IV iron (such as FCM 1000 mg) can safely improve Hb and energy levels and even help avoid transfusions in many cases [7].

Although the introduction of FCM in 2020 and FDI in 2023 provided an opportunity to adopt high-dose IV iron in oncology settings, real-world data are limited. The lack of ESAs as a treatment option necessitates an understanding of the efficacy of IV iron alone in Japanese patients with cancer and whether it can reduce transfusion risk. The limitations of oral iron further justify investigating IV iron as a primary therapy for CRA.

Therefore, this study aims to inform the optimal use of IV iron for CRA in Japan’s ESA-free context by providing real-world evidence on its (a) effectiveness, by evaluating high-dose IV iron (FCM or FDI) supplementation in Japanese patients with CRA, namely the Hb response at approximately 1 month post-infusion, proportion of patients achieving a meaningful Hb increase (≥1.0 g/dL), and rate of avoiding transfusions; (b) safety, by examining related outcomes with special attention to hypophosphatemia and infusion-related reactions; and (c) practical dosing considerations, by exploring a strategy aiming for approximately 1000 mg total iron repletion (either as a single 1000 mg FDI or 2 × 500 mg FCM dose infusions), assessing the adequacy of the administered dose relative to patients’ weight and calculated iron deficit (Ganzoni formula).

## 2. Materials and Methods

### 2.1. Study Design and Patients

This single-center retrospective cohort study conducted at a Japanese tertiary hospital included patients who received high-dose IV iron for CRA or CIA, between 1 October 2023 and 31 July 2025. Adult patients with solid or hematologic malignancies, CRA parameter of baseline Hb typically <10 g/dL with symptoms or <11 g/dL if plummeting, and routine care-administration of either FCM or FDI were included. Patients who received transfusions after IV iron were not excluded from the cohort; however, for certain efficacy analyses, subgroup or adjusted analyses were performed, as described below. All clinico-demographic data were obtained from the electronic medical records.

The Institutional Review Board of Toyama University approved the study (approval ID R2023041; 1 June 2023) and waived the requirement for individual informed consent (opt-out policy).

### 2.2. Treatment and Dosing

IV iron was prescribed at the discretion of the treating oncologists, generally for patients with confirmed or suspected ID. FCM was administered in 500 mg aliquots (maximum 500 mg/infusion, with some patients receiving two infusions approximately 1 week apart for a total of 1000 mg). FDI, introduced later in the study period, was typically administered as a single 1000 mg infusion. Each patient’s total iron dose was abstracted and estimated as mg/kg. Furthermore, each patient’s iron deficit was estimated using the Ganzoni formula (considering body weight, HB deficit to a target, and an allowance for iron stores approximately 500 mg) to assess dosing adequacy. Iron status was referentially categorized from laboratory data (if available) as absolute ID (ferritin < 100 ng/mL with TSAT < 20%), functional ID (TSAT < 20% with ferritin ≥ 100 ng/mL), or non-deficient/sufficient (TSAT ≥ 20% or ferritin above the needed thresholds).

### 2.3. Outcomes

The primary effectiveness outcome was change in Hb (ΔHb) from baseline to approximately 1 month post-infusion(s). The “1-month” Hb was defined as the closest Hb value in the 21–45 days post-infusion window (to allow for scheduling variances). Baseline Hb was defined as the last value pre-infusion (within the last 1 week). Patients without a follow-up Hb in the 21–45-day post-infusion window were excluded from the primary effectiveness analysis (available-case analysis without imputation). Secondary outcome included the responder rate, primarily, defined as the proportion of patients achieving a Hb increase of ≥1.0 g/dL, and, additionally, transfusion avoidance, defined as the percentage of patients who did not require any RBC transfusion between the index iron infusion and the 1-month assessment time-point. Safety outcomes included incidence of clinically significant hypophosphatemia, any infusion reactions, and other documented adverse events (e.g., skin staining or allergic reactions). Serum phosphate levels, at baseline and 2–6 weeks post-treatment to capture changes, were measured in a subset of patients.

### 2.4. Transfusion-Related Analyses

Since interim RBC transfusions can confound the assessment of Hb change attributable to IV iron, two prespecified approaches were used to handle transfused patients. First, primary efficacy analysis of ΔHb and responder rates was restricted to patients who received no RBC transfusions between the index iron infusion and the 1-month Hb measurement (thus analyzing a “pure” iron effect subgroup). Second, a sensitivity analysis was performed in the full cohort by adjusting the ΔHb for any transfusions. For each unit of RBC transfused, an imputed increment was subtracted from the observed ΔHb, using the following formula: 1.0 g/dL per unit × (70 kg/patient’s weight in kg) [11]. This assumes that one unit increases Hb by approximately 1 g/dL in a 70 kg adult, scaled to the patient’s size. Using this transfusion-adjusted ΔHb, the mean ΔHb and responder rates to estimate the “pure” iron effect. The unadjusted (transfusion-restricted) and adjusted analyses are both reported herein. We also performed a targeted chart review of patients who received transfusions (*n* = 10) to classify RBC transfusions occurring within 7 days after IV iron therapy (day 0–7) as prearranged (transfusion decision made before or on the day of iron administration) versus decided after IV iron therapy.

### 2.5. Statistical Analysis

Descriptive statistics was used to summarize patient characteristics and outcomes. Continuous variables (Hb and phosphate levels) are presented as mean ± standard deviation (SD) or median with interquartile range (IQR), as appropriate. Categorical variables (responder and transfusion avoidance rates) are presented as percentages. For within-patient change in Hb and phosphate levels, based on distribution, paired *t*-tests or Wilcoxon signed-rank tests were applied. The responder and transfusion avoidance rates are presented with 95% confidence intervals (CI; Clopper–Pearson method). The exploratory, retrospective nature of the study precluded an extensive hypothesis testing. A two-sided *p* < 0.05 was considered significant for the primary outcome of ΔHb. All analyses were conducted using available data without imputation for missing values. Python (version 3.11; Python Software Foundation) with pandas and SciPy libraries was used for data handling and statistical calculations.

Additional analyses were conducted in the primary effectiveness set using an available-case dataset with ~1-month Hb to explore dose–response patterns. Associations between ΔHb and (i) dose normalized by body weight (mg/kg) and (ii) Ganzoni coverage ratio (administered dose/Ganzoni-estimated iron need) were assessed using Spearman’s rank correlation and simple linear regression (Figure S3).

Effectiveness endpoints were further stratified by a curated three-level chemotherapy category (myelosuppressive, non-myelosuppressive, and no systemic therapy/best supportive care) and compared across groups using Kruskal–Wallis tests for continuous outcomes and Fisher’s exact tests for responder rates (Table S2). Within the functional iron deficiency phenotype, baseline predictors of response were explored using Mann–Whitney U tests and an exploratory multivariable logistic regression including CRP and albumin (Table S3).

## 3. Results

### 3.1. Cohort Characteristics

Of the 55 patients who met the inclusion criteria, 17 (31%) were female. The median age was 70 years (range, 34–82). Gastrointestinal and pancreatobiliary cancers predominated, followed by sarcomas and cancers of unknown primary. Baseline laboratory profiles confirmed a high prevalence of ID: 19 patients (34.5%) had absolute ID, 29 (52.7%) had functional ID, and 7 (12.7%) had non-deficiency or indeterminate ID. None of the patients received an ESA. The mean baseline Hb was 8.45 ± 1.48 g/dL, reflecting moderate-to-severe anemia, and 85% of patients had an Hb of <10 g/dL. Inflammatory markers (where available) were generally elevated (median CRP, 1.43 mg/dL), consistent with anemia of chronic disease.

Baseline cohort characteristics are summarized in Table 1.

### 3.2. IV Iron Treatment Patterns

FCM was the predominant formulation, and was administered in 52 patients. FDI was administered in 3 patients (all in the penultimate months of the study period after its approval). The total iron dose per patient was 500–1500 mg. While 34 (60%) patients received a single 500 mg infusion of FCM, 18 (33%) received two 500 mg FCM infusions approximately 1 week apart (total of 1000 mg), and 3 (5%) received a single 1000 mg FDI infusion. Thus, 38.2% of the cohort achieved a cumulative dose of ≥1000 mg, whereas the remainder received <1000 mg. In weight-adjusted terms, the median dose was 10.4 mg of elemental iron per kg body weight (IQR, 8.8–18.3 mg/kg). Comparison of the administered doses to each patient’s estimated total iron deficit (Ganzoni formula) revealed that the median calculated need was 972 mg (IQR, 830–1097 mg). The median delivered iron-to-required iron (“coverage”) ratio was 0.72 (IQR, 0.48–0.91), indicating that most patients under-dosed relative to full repletion, i.e., approximately 72% of the estimated requirement. Only 20 (36%) patients received iron equaling or exceeding their calculated deficit. These findings highlight a cautious dosing in practice: many patients did not receive the guideline-recommended approximately 1000 mg if their weight or Hb deficit would have justified it, often because of the common practice of administering a single 500 mg FCM infusion as a standard initial dose.

Key dosing metrics and effectiveness readouts (approximately 1 month) are summarized in Table 2.

### 3.3. Hemoglobin Response

Among the non-transfused patients (*n* = 45, transfusion details elucidated below), Hb increased from 8.76 ± 1.34 g/dL at baseline to 9.73 ± 1.75 g/dL approximately 1 month post-infusion. The mean ΔHb was +0.92 g/dL (SD, 1.44; *p* < 0.001), which was clinically meaningful. More than 50% of the patients achieved a notable Hb increase: the responder rate was 48.9% (22/45 patients; 95% CI, 39–66%). While 24% of the patients had an Hb increase of ≥2 g/dL, 33% had marginal to no Hb increase (<0.5 g/dL), appearing to correlate with the values of those who received lower iron doses relative to need or had ongoing bleeding.

**Figure 1:**
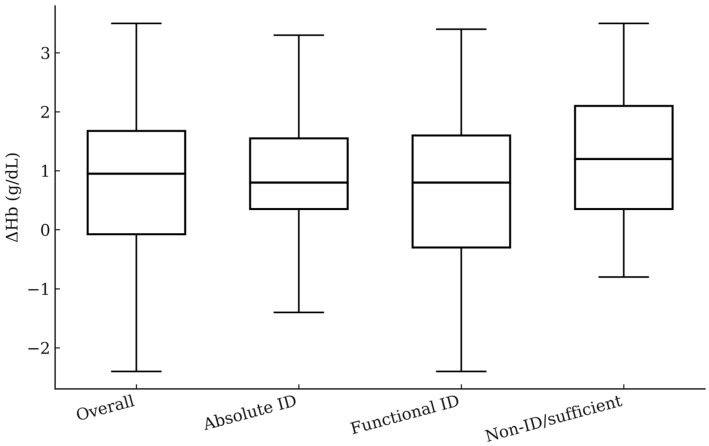
Change in hemoglobin (ΔHb) at ~1 month by iron status (non-transfused set). Box = interquartile range (IQR); center line = median; whiskers = 1.5 × IQR; outliers are points beyond the whiskers. “~1 month” denotes the latest Hb measured 21–45 days after iron.

The distribution of ΔHb overall and by iron status in the non-transfused set is illustrated in Figure 1; Hb distributions by iron status at baseline and approximately 1-month post-infusion are presented in Figure S1.

In exploratory dose–response analyses among patients with available ΔHb data (*n* = 44), ΔHb showed weak inverse associations with both the dose normalized by body weight (mg/kg) and Ganzoni coverage ratio (administered dose/Ganzoni-estimated iron need) (Spearman ρ = −0.27, *p* = 0.077; ρ = −0.29, *p* = 0.056, respectively; Figure S3). In simple linear regression, the slope for ΔHb versus dose was −0.05 g/dL per 1 mg/kg (95% CI −0.12 to 0.02; *p* = 0.137), and the slope versus Ganzoni coverage was −0.15 g/dL per 0.1 increase (95% CI −0.30 to 0.01; *p* = 0.060).

When stratified by chemotherapy category (Table S2), the median ΔHb was 0.90 g/dL in the myelosuppressive group (*n* = 25), 2.20 g/dL in the non-myelosuppressive group (*n* = 8), and 0.00 g/dL in the no systemic therapy/best supportive care group (*n* = 11 with ~1-month Hb); responder rates (ΔHb ≥ 1.0 g/dL) were 48.0%, 62.5%, and 45.5%, respectively, without significant differences across the groups (*p* = 0.261 for ΔHb; *p* = 0.840 for responders).

Within the functional iron deficiency phenotype, baseline CRP, albumin, ferritin, and TSAT did not differ between responders and non-responders (all *p* ≥ 0.573; Table S3). In an exploratory logistic regression including CRP and albumin (*n* = 26), neither variable was associated with response (CRP OR 0.92 per 1 mg/dL, 95% CI 0.77–1.09, *p* = 0.338; albumin OR 0.87 per 1 g/dL, 95% CI 0.21–3.72, *p* = 0.856).

For the transfusion-adjusted analysis, only 10 patients (18.2%) received RBC transfusion after IV iron therapy and were included. After subtracting the estimated transfusion contributions to Hb, the adjusted mean ΔHb attributable to IV iron ranged between +1.1 to +1.3 g/dL, and the adjusted responder rate remained unchanged (~50% achieved an Hb increase of ≥1.0 g/dL), indicating that the overall effectiveness signal was robust. Transfusion timing relative to IV iron therapy was evaluable in 9 of 10 patients who received transfusions; eight (88.9%) received RBC transfusion within 7 days of IV iron therapy (days 0–7). Among these early transfusions (*n* = 8), four (50.0%) were pre-arranged based on clinical assessment before or on the day of iron administration, whereas four (50.0%) were decided after IV iron therapy because of clinical urgency before a hematologic response could reasonably be expected (baseline Hb in the early-transfusion group: median 6.8 g/dL [IQR 6.4–7.7]; transfusion volume: median 3 units [IQR 2–5], total 30 units). Thus, early transfusions should be interpreted as reflecting baseline severity and clinical urgency rather than an early sign of IV-iron treatment failure.

### 3.4. Transfusion Avoidance

Overall, 45 of 55 patients (transfusion avoidance rate, 81.8%) required no RBC transfusions following IV iron administration. Among the 10 patients who received transfusions, the timing relative to IV iron therapy was evaluable in nine; eight (88.9%) occurred within 7 days (days 0–7), including four pre-arranged transfusions (decision made before or on the day of iron administration) and four transfusions decided after IV iron therapy because of clinical urgency. These early transfusions likely reflect severe baseline anemia and an urgent need for symptom control before an Hb response to IV iron therapy could be expected, rather than an early lack of treatment effectiveness.

The observed transfusion avoidance rate should be interpreted cautiously, as this study lacks a control group and transfusion decisions are influenced by baseline severity, symptoms, and institutional practices. Without a comparator, we cannot definitively quantify the extent to which transfusions were prevented by IV iron therapy. However, compared with the historical practice in Japan (where, in an ESA-free setting, a substantial fraction of patients with Hb < 8–9 g/dL might eventually require transfusion [8], these findings suggest that IV iron therapy may offer a practical supportive-care option for selected patients with moderate anemia who can be safely observed.

### 3.5. Serum Iron and Ferritin Changes

Iron assessments were not uniformly repeated post-infusion in all patients; nevertheless, expected changes were observed in those with available data, confirming the successful delivery and utilization of iron to replenish stores. Ferritin levels increased substantially in patients who received near-total iron repletion. In the absolute ID group, median ferritin increased from approximately 30 ng/mL at baseline to approximately 120 ng/mL at 4–6 weeks post-treatment in those who received ≥1000 mg. Moreover, TSAT values (when checked) improved from a median of approximately 10% at baseline to 20–25% post-treatment. In the functional ID group, baseline ferritin was higher (often 200–400 ng/mL with low TSAT). Although ferritin did not change markedly with iron (some even decreased as iron was incorporated into erythropoiesis), TSAT trended toward normal in responders. Post-treatment iron assessment was not the focus of our analysis; however, it indicates that dosing was sufficient to correct absolute ID, albeit not enough to replenish stores in functional ID.

### 3.6. Safety Outcomes

IV iron was generally well-tolerated. No acute infusion reactions were observed. Specifically, no cases of anaphylaxis or clinically significant hypotension were noted during or post-infusion. Furthermore, no development of permanent skin staining at injection sites was reported; the infusions were administered with appropriate technique, and no extravasation injuries occurred.

Regarding hypophosphatemia, a known potential side effect of high-dose IV iron (especially FCM), serum phosphate was measured in 46 patients (84%) at baseline and approximately 1-month post-infusion. Median phosphate decreased from 3.4 [3.0–3.9] to 3.2 [2.7–3.8] mg/dL. While 10 patients (22%) had biochemical hypophosphatemia with values < 2.5 mg/dL, including 7 (15%) with values < 2.0 mg/dL, none were symptomatic or required intervention. These findings indicate a low incidence of clinically relevant hypophosphatemia; however, phosphate nadir may have occurred earlier (2–3 weeks post-infusion) and could have been missed in some cases owing to laboratory assessment timing. Nevertheless, the lack of any severe hypophosphatemia is reassuring, considering literature reports of higher incidence with FCM. No other IV iron-related adverse effects, such as cardiac or infectious safety signals, were observed within 1 month.

Pre-treatment and approximately 1-month post-treatment serum phosphate values are summarized in Table 3. Other zero-event safety signals (symptomatic hypophosphatemia, infusion reactions, and skin staining) have been reported in the text.

## 4. Discussion

In this single-center retrospective cohort from Japan, where ESAs are not approved for chemotherapy-induced anemia, high-dose IV iron monotherapy (FCM or FDI) was associated with short-term Hb improvement in a subset of patients, with a high observed rate of transfusion avoidance over a 21–45-day follow-up window and no serious treatment-emergent safety signals. Given the modest sample size, observational design, and limited follow-up, these findings should be interpreted as context-specific and hypothesis-generating rather than as definitive evidence of efficacy or broad generalizability.

### 4.1. Principal Findings

High-dose IV iron therapy without ESA support was associated with a clinically meaningful rise in Hb on average at approximately 1 month (21–45 days), with about half of patients meeting the prespecified responder definition and most patients not receiving RBC transfusion during this short observation period. In the Japanese setting where ESAs are not used for chemotherapy-induced anemia, our clinical aim is typically not Hb normalization but rather stabilization of anemia and potential avoidance of near-term transfusion when feasible. No infusion reactions or symptomatic hypophosphatemia were documented, supporting the short-term tolerability of modern high-dose IV iron therapy in routine oncology practice.

Our observations are broadly consistent with those of prior reports that IV iron therapy can improve Hb in selected patients with CRA/CIA, including studies evaluating high-dose FCM without ESA rescue [7,12]. However, direct comparisons are limited by differences in study design, patient selection, and assessment timepoints. Because our primary endpoint was defined within a 21–45-day window, we cannot determine the durability of the Hb response beyond the first month or establish longer-term management strategies.

The transfusion avoidance rate observed in our cohort over approximately 1 month is encouraging, but it should be interpreted cautiously because we lacked a control group and transfusion decisions are influenced by baseline severity, symptoms, and institutional practices. Notably, most post-infusion transfusions occurred within 7 days, often as pre-arranged or prompted by clinical urgency, before a hematologic response to iron therapy would be expected. Thus, early transfusion should not be interpreted as a marker of IV-iron nonresponse. Rather, our findings suggest that, for patients with moderate anemia who can be safely observed, IV iron therapy may offer a practical supportive-care option to stabilize Hb in the short term, while acknowledging that some patients will still require transfusion for symptomatic or severe anemia.

### 4.2. Comparison with Guidelines and Literature

International guidelines emphasize assessing the iron status in CRA and correcting iron deficiency, and ESMO guidance includes consideration of high-dose IV iron therapy (e.g., 1000 mg) for functional iron deficiency in patients with Hb in the 8–10 g/dL range [2]. In our cohort, patients generally fell within similar laboratory and Hb ranges, and our short-term results are directionally consistent with the concept that iron repletion can improve anemia in some patients. Many patients in routine practice received less than guideline-referenced repletion doses, reflecting cautious real-world dosing. These findings highlight variability in practice and the potential value of standardizing assessment (e.g., TSAT-based selection) and reassessment after an initial course.

From an international perspective, in settings where ESAs are available, IV iron is typically used as an adjunct (alone or combined with ESAs) to support erythropoiesis and reduce transfusion requirements [4,6]. Our ESA-free cohort represents a different clinical context in which IV iron therapy is often one of few disease-modifying options available before transfusion. The responder rate in our study suggests that iron repletion alone may be sufficient for early Hb improvement in a subset of patients, whereas others may likely have multifactorial anemia not be fully addressed by iron alone. Accordingly, our findings should not be interpreted as advocating IV iron therapy as a universal substitute for ESA-based strategies, but rather as suggesting a pragmatic option in an ESA-unavailable setting.

### 4.3. Hypophosphatemia and Safety Considerations

Fibroblast growth factor 23 (FGF23)-induced hypophosphatemia is a key safety concern with high-dose IV iron [13,14]. Mechanistically, FCM increases FGF23, which promotes renal phosphate excretion [13], whereas FDI’s molecular structure retards iron release, causing less FGF23 rise and consequently lower hypophosphatemia incidence [14]. A trial reported transient hypophosphatemia (phosphate < 2 mg/dL) in 50–75% and <10% of patients receiving FCM and FDI, respectively [15].

In contrast, our data indicated no severe or symptomatic hypophosphatemia: only mild, transient phosphate declines were observed in a few patients. Several factors may explain this difference. First, our sample size is modest, and phosphate was not monitored at the nadir time for all patients (2–3 weeks post-infusion for FCM [16]), leading to possible asymptomatic hypophosphatemia under-detection—a limitation noted by Reviewer 1. Second, many of our patients received 500 mg FCM rather than a single 1000 mg dose; smaller divided doses might lessen the FGF23 surge and phosphate decline. Third, the few patients who received a single 1000 mg iron dose were treated with FDI, which has a significantly lower risk of hypophosphatemia than FCM [16,17].

A Japanese trial reported severe hypophosphatemia in 0% of FDI-treated patients vs. approximately 7% of low-dose SFO-treated patients [10]. Clinicians should be aware that SFO induces FGF23-mediated phosphate wasting similar to FCM [14]. Patients with risk factors such as low vitamin D or borderline baseline phosphate levels, or those needing multiple iron courses might require phosphate monitoring and supplementation. FDI might be preferable for patients requiring frequent iron or those at risk for osteopenia. None of the patients in our cohort developed osteomalacia or musculoskeletal symptoms, which is a rare occurrence with repeated FCM in other settings [13].

Overall, our safety profile for IV iron was excellent. No anaphylactic or hypotensive reactions were observed in our cohort (FCM, *n* = 52 and FDI, *n* = 3), aligning with the known <1:1000 risk of serious reactions for FCM and FDI reported in the literature [18]. These findings should reassure clinicians that FCM or FDI can safely be administered in oncology settings, even in patients with frailty or those receiving concurrent chemotherapy.

### 4.4. Dosing Adequacy and Practical Implications

Our study spotlights the gap between guideline-referenced iron dosing and real-world practice. Although guidelines commonly reference a 1000-mg course for most patients with ID [2], many patients received lower doses. In exploratory, uncontrolled comparisons, patients who received approximately 1000 mg (or more) and 500 mg exhibited a mean Hb increase of 1.8 g/dL and 0.7 g/dL, respectively; however, these observations are susceptible to confounding and should be interpreted as hypothesis-generating rather than as prescriptive.

We did not observe a clear positive dose–response relationship between ΔHb and either the mg/kg dose or Ganzoni coverage (Figure S3), which may reflect confounding by indication. Stratification by chemotherapy category suggested that Hb improvements were not confined to a specific treatment intensity group, and baseline inflammatory/nutritional markers did not clearly identify responders within functional iron deficiency categories (Tables S2 and S3). These findings support a pragmatic approach that emphasizes achieving guideline-consistent iron repletion while acknowledging heterogeneity in CRA.

### 4.5. Relation to Recent Japanese Data

A recent single-center retrospective study in Japan evaluated an IV-iron group (single 1000-mg FDI course) versus a conventional non-IV-iron control in a gynecologic oncology cohort [18]. By day 21, Hb increased from 7.6 to 9.4 g/dL in the IV-iron group, and RBC transfusion was required in 7% compared with 33% in controls. While broadly consistent with our short-term signals, that study differed in scope and did not classify ID phenotypes. Together, these early Japanese real-world reports suggest that IV-iron monotherapy can be considered as a supportive-care approach in ESA-unavailable settings, but both were observational and short-term.

### 4.6. Strengths and Limitations

Some limitations of this study warrant emphasis. First, this was a retrospective, single-center, uncontrolled study; treatment selection, supportive-care co-interventions, cancer trajectory, and chemotherapy intensity may confound observed Hb changes, and causal attribution to IV iron therapy is not possible. Second, the single-center drawn modest sample size (*N* = 55) limits generalizability and subgroup analyses (e.g., comparing tumor types or FCM vs. FDI abreast). Notably, all 3 FDI-treated patients responded well without hypophosphatemia; however, the number is negligible to definitively conclude on the between-treatment efficacy differences. Therefore, observations related to FDI should be interpreted as exploratory and descriptive given the very small sample size (*n* = 3), and the overall conclusions of this study are driven primarily by the much larger FCM cohort. As FDI use expands, future studies should examine if its efficacy matches that of FCM, thus confirming its low rate of hypophosphatemia in oncology settings. Third, outcomes were assessed within a prespecified short window (21–45 days), capturing an initial treatment course but precluding conclusions regarding the durability of the Hb response, repeat dosing or maintenance strategies, or longer-term safety, including cumulative risk of hypophosphatemia with repeated courses. Fourth, the mean Hb values remained below 10 g/dL in many patients; our data therefore do not support Hb normalization as a realistic expectation of IV iron monotherapy in this setting. Transfusion thresholds vary across institutions, and without a comparator we cannot quantify the extent to which transfusions were prevented. Finally, laboratory monitoring (post-treatment iron indices and the timing of phosphate measurements) and patient-reported outcomes were not uniformly available.

### 4.7. Future Directions and Implementation

Future work should prioritize larger, multicenter prospective studies in Japan and other practice settings to confirm these short-term findings, evaluate the durability of the response beyond 1 month, and define pragmatic re-dosing/maintenance strategies. Comparative studies of FCM versus FDI in oncology populations with systematic phosphate monitoring would clarify formulation-specific safety profiles, particularly with repeated courses.

As an operational tool for our institution, we developed a TSAT-guided pathway for patient selection and dosing aiming to deliver an initial high-dose course (typically targeting ~1000 mg total iron) with reassessment at approximately 4 weeks (Figure S2). We present this pathway as a practical example of how IV iron therapy can be integrated into routine care in an ESA-unavailable setting, not as a guideline or an implementation recommendation. Looking ahead, emerging anemia therapies (e.g., hypoxia-inducible factor prolyl hydroxylase inhibitors or hepcidin-targeting strategies) may expand options for patients who do not adequately respond to iron alone. In the meantime, careful identification of iron deficiency and pragmatic use of IV iron therapy remain reasonable supportive-care considerations in Japan.

## 5. Conclusions

This retrospective single-center study suggests that, in a Japanese oncology setting, where ESAs are not available for chemotherapy-induced anemia, high-dose IV iron monotherapy (FCM or FDI) may offer a pragmatic supportive-care option for selected patients with iron-restricted CRA/CIA. Within a 21–45-day assessment window, IV iron was well-tolerated and was associated with modest Hb improvement in a subset of patients with a high observed rate of transfusion avoidance. These findings should not be generalized beyond this context or interpreted as evidence for long-term Hb maintenance or specific re-dosing strategies; confirmation in larger studies with longer follow-up and standardized symptom and safety monitoring is needed. In regions where ESAs are available, IV iron therapy may be viewed as complementary rather than as substitutive, supporting guideline-based anemia management.

## Figures and Tables

**Table 1:** Baseline cohort characteristics.

Parameter	Overall (*N* = 55)
Demographics and anthropometrics	
Age, years	70.0 [61.0–76.0]
Sex, *n* (%)	
Male	38 (69.1)
Female	17 (30.9)
Weight, kg	54.2 [47.9–61.5]
Baseline laboratory values	
C-reactive protein, mg/dL	1.43 [0.54–5.48]
Albumin, g/dL	3.3 [2.8–3.6]
Transferrin saturation, %	12.2 [8.0–18.2]
Ferritin, ng/mL	187 [27–324]
Serum iron, µg/dL	32 [19–51]
Cancer type, *n* (%)	
Gastrointestinal	23 (41.8)
Pancreatobiliary	18 (32.7)
Other	10 (18.2)
Sarcoma	4 (7.3)
Treatment status at iron administration, *n* (%)	
On chemotherapy	40 (72.7)
Best supportive care only (non-chemotherapy)	15 (27.3)
Iron phenotype, *n* (%)	
Absolute iron deficiency ^a^	19 (34.5)
Functional iron deficiency ^b^	29 (52.7)
Non-deficient/sufficient	7 (12.7)
Documented bleeding etiology, *n* (%)	
None documented	48 (87.3)
Primary tumor	5 (9.1)
Diverticular	1 (1.8)
Duodenal ulcer	1 (1.8)

Values are median [IQR] for continuous variables and *n* (%) for categorical variables. Percentages are based on non-missing data. ^a^ Absolute iron deficiency: ferritin < 100 ng/mL with TSAT < 20%. ^b^ Functional iron deficiency: TSAT < 20% with ferritin ≥ 100 ng/mL.

**Table 2:** Key dosing and effectiveness outcomes (approximately 1 month).

Outcome	Definition/Analysis Set	Value
Cohort size (available-case)	Retrospective single-center cohort	*N* = 55
Effectiveness outcomes (~1 month)		
Responder rate	ΔHb ≥ 1.0 g/dL; excludes any RBC transfusion within 21–45 days (non-transfused set)	48.9%, *n* = 45
Transfusion avoidance	0 RBC units from index infusion to approximately 1 month (21–45 days); full cohort	81.8%
Dosing adequacy metrics		
1000 mg attainment	FDI 1000 mg × 1 OR FCM 500 mg × 2	38.2%
Dose per person	Administered dose/body weight; median [IQR]	10.4 [8.8–18.3] mg/kg
Ganzoni iron need	Estimated iron deficit; median [IQR]	972 [830–1097] mg
Dose coverage	Administered/Ganzoni need; median [IQR]	0.72 [0.48–0.91]
Hb summary (non-transfused set)		
Baseline Hb	Mean ± SD	8.76 ± 1.34 g/dL
1-month Hb	Mean ± SD; window 21–45 days	9.73 ± 1.75 g/dL
ΔHb	Mean ± SD	+0.92 ± 1.44 g/dL

Abbreviations: FCM, ferric carboxymaltose; FDI, ferric derisomaltose; Hb, hemoglobin; RBC, red blood cell. “1 month” = closest hemoglobin within 21–45 days post-infusion.

**Table 3:** Serum phosphate (safety summary).

Time-Point	Serum Phosphate, mg/dL (Median [IQR])
Pre-treatment	3.4 [3.0–3.9]
~1 month post-treatment	3.2 [2.7–3.8]

## Data Availability

De-identified data for this study are stored on secure institutional servers at Toyama University Hospital. Owing to institutional policies and privacy restrictions, the data are not publicly accessible. Requests to access additional de-identified data should be directed to the corresponding author. Data will be made available upon reasonable request, subject to a data-sharing agreement and approval from the Institutional Review Board of Toyama University.

## Supplementary Materials

The following supporting information can be downloaded at: https://www.mdpi.com/article/10.3390/cancers18030416/s1, Figure S1: Distribution of hemoglobin at baseline and at ~1 month by iron status; Figure S2: TSAT-guided clinical pathway for cancer-related anemia (Toyama University Hospital); Figure S3: Dose—response analyses (ΔHb vs. dose metrics); Table S1: Analysis sets, dose metrics, and missingness; Table S2: Stratified effectiveness by chemotherapy category; Table S3: Predictors of response within functional iron deficiency. Table S4. Timing and clinical context of RBC transfusions after IV iron.

## Abbreviations

The following abbreviations are used in this manuscript:

CRA	Cancer-related anemia
ESA	Erythropoiesis-stimulating agent
IV	Intravenous
FCM	Ferric carboxymaltose
FDI	Ferric derisomaltose
ID	Iron deficiency
TSAT	Transferrin saturation
Hb	Hemoglobin
CRP	C-reactive protein
QoL	Quality of life
CIA	Chemotherapy-induced anemia
ESMO	European Society for Medical Oncology
RBC	Red blood cell
SD	Standard deviation
IQR	Interquartile range
SFO	Saccharated ferric oxide
FGF23	Fibroblast growth factor 23
HIF-PH	Hypoxia-inducible factor prolyl hydroxylase
