# Supplementary material for: High-Dose Intravenous Ferric Carboxymaltose/Derisomaltose Without ESAs for Cancer-Related Anemia in Japan: A Retrospective Single-Center Cohort Study
Source: Cancers (Basel). 2026 Jan 28;18(3):416. doi: 10.3390/cancers18030416 (PMC12896494; doi:10.3390/cancers18030416)
Supplement: Supplementary file 1 [file cancers-18-00416-s001.zip › cancers-4052478-supplementary.pdf]

## Supplementary Materials

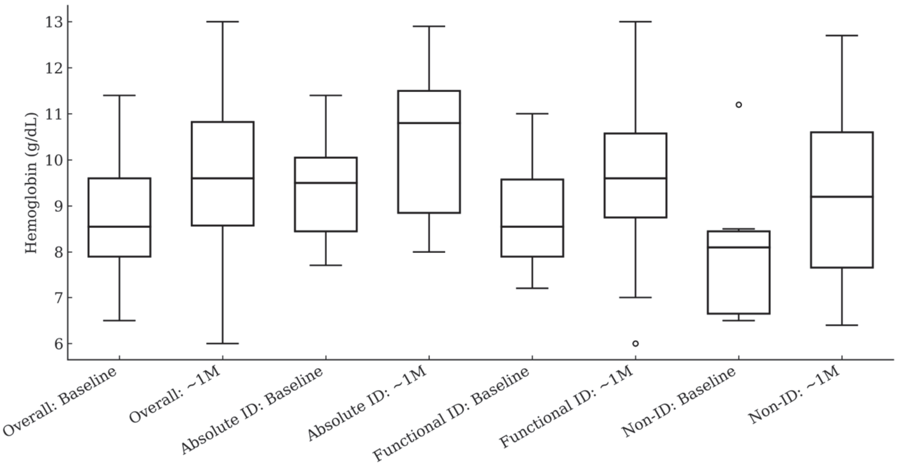


**Figure S1. Distribution of hemoglobin at baseline and at ~1 month by iron status.**

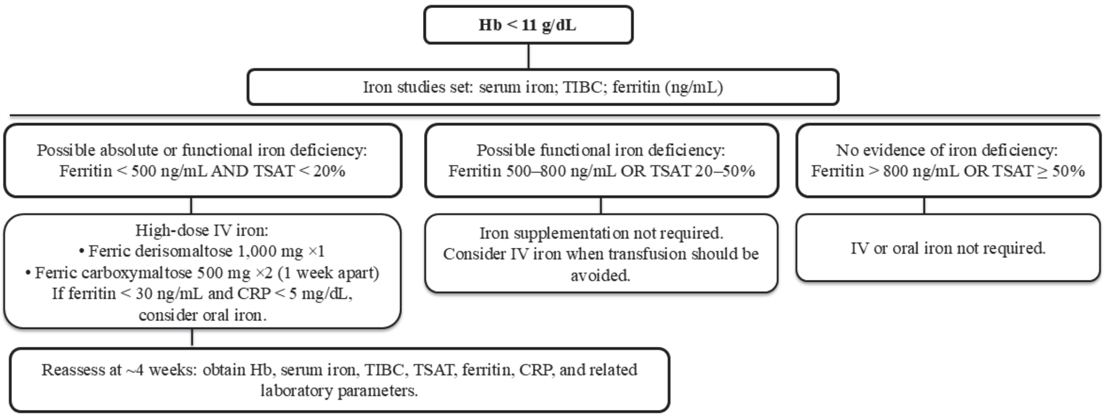


* TIBC = total iron-binding capacity; TSAT = transferrin saturation (serum iron/TIBC).

**Figure S2. TSAT-guided clinical pathway for cancer-related anemia (Toyama University Hospital).**

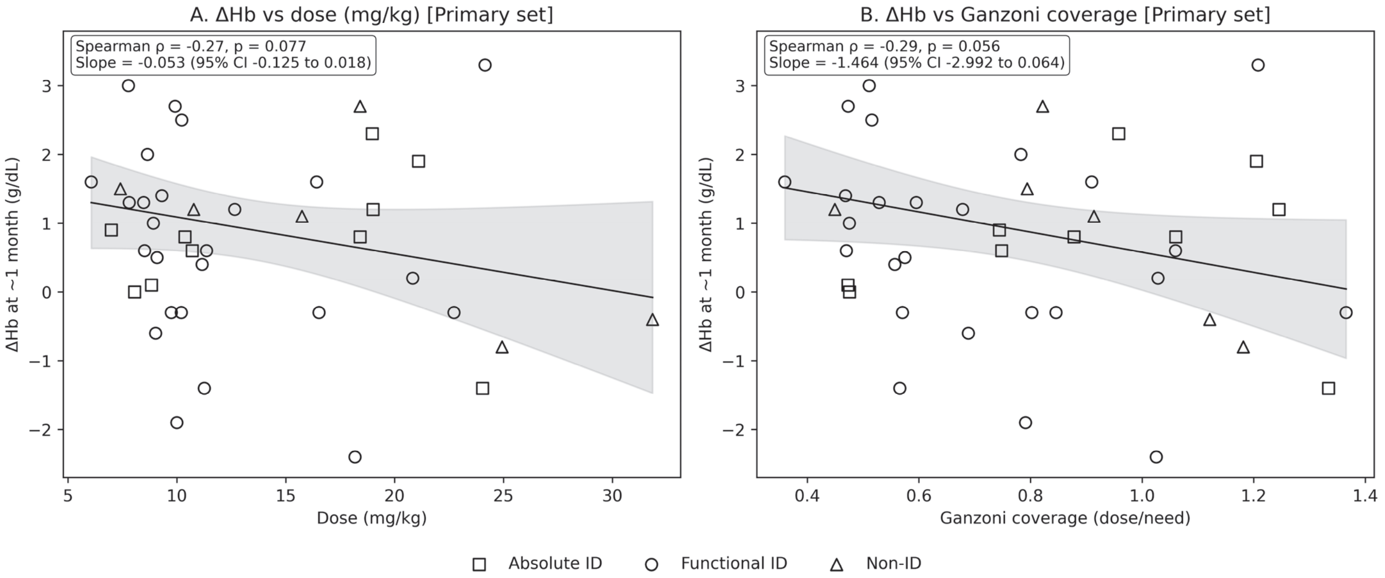


**Figure S3.** Dose-response analyses (Primary effectiveness set).
Panel A: ΔHb vs dose (mg/kg). Panel B: ΔHb vs Ganzoni coverage ratio (dose/need). Primary set n=45; available-case for ΔHb n=44.
Figure notes: Spearman ϱ and p-values; linear regression slope (95% CI) shown in each panel. Points are shaped by ID phenotype (Absolute/Functional/Non-ID).

**Table S1.** Analysis sets, dose metrics, and missingness.

| Item | Value (Primary set centered) | Notes |
|---|---|---|
| Analysis sets (n) | | |
| Full cohort (all patients) | 55 | All rows in Excel |
| Primary effectiveness set (non-transfused subset) | 45 | transfusion_units = 0 |
| Primary set with ~1M Hb available (for ΔHb/responder/dose-response) | 44 | available-case (Hb at ~1M not missing) |
| ID phenotype in Primary set: Absolute ID | 11 | Baseline definition: TSAT and ferritin |
| ID phenotype in Primary set: Functional ID | 27 | Baseline definition: TSAT and ferritin |
| ID phenotype in Primary set: Non-ID | 7 | Baseline definition: TSAT and ferritin |
| Functional ID subset within Primary set | 27 (total), 26 (with ~1M Hb) | Used for Table S3 |
| Dose metrics in Primary set (n=45) | Median [IQR] (Mean ± SD) | |
| Total iron dose (mg) | 500.00 [500.00–1000.00] (688.89 ± 245.16) | |
| Body weight (kg) | 54.00 [48.00–59.00] (54.46 ± 10.80) | |
| Dose (mg/kg) | 10.37 [8.83–18.42] (13.32 ± 6.12) | |
| Ganzoni need (mg) | 943.09 [802.98–1056.94] (919.38 ± 187.11) | |

| | | |
|---|---|---|
| Ganzoni coverage (dose/need) | 0.75 [0.52–0.96] (0.77 ± 0.28) | |
| Dose ≥1000 mg, n (%) | 17/45 (37.8%) | |
| | | |
| Missingness in Primary set (n=45) | n missing | available-case per analysis |
| Hb at ~1M missing | 1 | |
| ΔHb missing | 1 | |
| Body weight missing | 0 | |
| Ganzoni need missing | 0 | |
| Dose mg/kg missing | 0 | |
| CRP missing | 0 | |
| Albumin missing | 0 | |
| Ferritin missing | 0 | |
| TSAT missing | 0 | |
| | | |
| Chemotherapy category counts (n) | | Curated 3-level classification; no recategorization |
| Myelosuppressive | 25 | |
| Non-myelosuppressive | 8 | |
| No systemic / BSC | 12 | |

**Table S2.** Stratified effectiveness by chemotherapy category (Primary set).

| Chemotherapy category | n (Primary) | n with ~1M Hb | Baseline Hb (g/dL), median [IQR] | ~1M Hb (g/dL), median [IQR] | ΔHb (g/dL), median [IQR] | ΔHb (g/dL), mean ± SD | Responder (ΔHb≥1.0), n/N (%) |
|---|---|---|---|---|---|---|---|
| Myelosuppressive | 25 | 25 | 8.30 [7.70–9.60] | 9.20 [8.70–10.50] | 0.90 [0.40–1.50] | 0.86 ± 1.23 | 12/25 (48.0%) |
| Non-myelosuppressive | 8 | 8 | 8.60 [8.30–8.95] | 11.05 [9.90–11.93] | 2.20 [0.50–3.08] | 1.57 ± 2.01 | 5/8 (62.5%) |
| No systemic / BSC | 12 | 11 | 9.30 [8.03–9.93] | 9.70 [8.40–10.65] | 0.00 [-0.30–1.30] | 0.57 ± 1.39 | 5/11 (45.5%) |
| p-value (across 3 groups) | | | 0.685 | 0.357 | 0.261 | | 0.840 |

**Table S3.** Predictors of response within Functional ID (Primary set).

| Variable (baseline) | Responder (ΔHb≥1.0), median [IQR] | Non-responder, median [IQR] | p (Mann–Whitney U) | |
|---|---|---|---|---|
| CRP (mg/dL) | 2.02 [1.18–5.43] | 2.71 [1.03–8.09] | 0.720 | |
| Albumin (g/dL) | 3.10 [2.60–3.60] | 2.80 [2.60–3.40] | 0.643 | |
| Ferritin (ng/mL) | 275.00 [210.00–404.00] | 298.00 [271.00–396.00] | 0.573 | |
| TSAT (%) | 13.87 [11.59–15.93] | 14.04 [8.82–18.08] | 0.959 | |
| **Predictor** | **OR** | **95% CI lower** | **95% CI upper** | **p** |
| CRP (per 1 mg/dL) | 0.9177973401921463 | 0.7700331607924343 | 1.0939164708139602 | 0.3381980821973072 |

| | | | | |
|---|---|---|---|---|
| Albumin (per 1 g/dL) | 0.874000780395911 | 0.20508464548366195 | 3.7246931008958235 | 0.8555186470410754 |

**Table S4.** Timing and clinical context of RBC transfusions after IV iron.

| Case | Days from IV iron to transfusion | Decision context | RBC units |
|---|---|---|---|
| 1 | 0 | Pre-arranged (decision before/same day as IV iron) | 6 |
| 2 | 0 | Pre-arranged (decision before/same day as IV iron) | 2 |
| 3 | 0 | Pre-arranged (decision before/same day as IV iron) | 2 |
| 4 | 1 | Pre-arranged (decision before/same day as IV iron) | 8 |
| 5 | 2 | Decided after IV iron | 4 |
| 6 | 3 | Decided after IV iron | 2 |
| 7 | 5 | Decided after IV iron | 4 |
| 8 | 7 | Decided after IV iron | 2 |
| 9 | 26 | Decided after IV iron | 2 |
| 10 | NA | Not documented | 2 |

Notes: Day 0 indicates the date of high-dose IV iron initiation. 'Pre-arranged' indicates that the decision to transfuse was made before or on day 0, whereas 'Decided after IV iron' indicates that the decision was made after iron administration. One case lacked a documented transfusion date in the medical record.
